# Case report: Neural timing deficits prevalent in developmental disorders, aging, and concussions remediated rapidly by movement discrimination exercises

**DOI:** 10.3389/fneur.2023.898781

**Published:** 2023-09-25

**Authors:** Teri Lawton, John Shelley-Tremblay, Ming-Xiong Huang

**Affiliations:** ^1^Cognitive Neuroscience, Perception Dynamics Institute, Encinitas, CA, United States; ^2^Department of Psychology, University of South Alabama, Mobile, AL, United States; ^3^Radiology Imaging Laboratory, Department of Radiology, University of California, San Diego, San Diego, CA, United States

**Keywords:** timing deficits, magnocellular deficits, cognitive remediation, cortical plasticity, reading/attention/memory/executive control networks, perceptual learning

## Abstract

**Background:**

The substantial evidence that neural timing deficits are prevalent in developmental disorders, aging, and concussions resulting from a Traumatic Brain Injury (TBI) is presented.

**Objective:**

When these timing deficits are remediated using low-level movement-discrimination training, then high-level cognitive skills, including reading, attention, processing speed, problem solving, and working memory improve rapidly and effectively.

**Methods:**

In addition to the substantial evidence published previously, new evidence based on a neural correlate, MagnetoEncephalography physiological recordings, on an adult dyslexic, and neuropsychological tests on this dyslexic subject and an older adult were measured before and after 8-weeks of contrast sensitivity-based left–right movement-discrimination exercises were completed.

**Results:**

The neuropsychological tests found large improvements in reading, selective and sustained attention, processing speed, working memory, and problem-solving skills, never before found after such a short period of training. Moreover, these improvements were found 4 years later for older adult. Substantial MEG signal increases in visual Motion, Attention, and Memory/Executive Control Networks were observed following training on contrast sensitivity-based left–right movement-discrimination. Improving the function of magnocells using figure/ground movement-discrimination at both low and high levels in dorsal stream: (1) improved both feedforward and feedback pathways to modulate attention by enhancing coupled theta/gamma and alpha/gamma oscillations, (2) is adaptive, and (3) incorporated cycles of feedback and reward at multiple levels.

**Conclusion:**

What emerges from multiple studies is the essential role of timing deficits in the dorsal stream that are prevalent in developmental disorders like dyslexia, in aging, and following a TBI. Training visual dorsal stream function at low levels significantly improved high-level cognitive functions, including processing speed, selective and sustained attention, both auditory and visual working memory, problem solving, and reading fluency. A paradigm shift for treating cognitive impairments in developmental disorders, aging, and concussions is crucial. Remediating the neural timing deficits of low-level dorsal pathways, thereby improving both feedforward and feedback pathways, before cognitive exercises to improve specific cognitive skills provides the most rapid and effective methods to improve cognitive skills. Moreover, this adaptive training with substantial feedback shows cognitive transfer to tasks not trained on, significantly improving a person’s quality of life rapidly and effectively.

## 1. Introduction

The brain needs to orchestrate and integrate the activity of different cortical areas that are involved in a particular task. This is accomplished by boosting synchronized oscillations that occur between these cortical areas. The neural timing deficits that are found in a wide range of brain disorders, affecting these synchronized oscillations are not well understood. Much evidence has now accumulated to suggest that a fundamental deficit in developmental dyslexia (1–5) atypical, in older adults (6–10), and in traumatic brain injury (TBI) (11–13) is impaired operation of the visual timing functions mediated by the magnocellular system (14). Moreover, this review concludes: “These studies suggest that a paradigm shift from phonologically-based to visually-based methods is required for the treatment of dyslexia. In older adults and following a concussion, the same paradigm shift is also called for. Moreover, this adaptive training, with substantial feedback and rewards, shows cognitive transfer to tasks not trained and can thus help to improve a person’s quality of life rapidly and effectively. The critical issue is that regardless of input modality, effective treatments must address neural timing deficits.”

We will describe additional evidence showing that neural timing deficits that are prevalent in many different types of cognitive impairments, including developmental disorders, like dyslexia where reading is difficult, normal aging, and concussions, reported previously (15), are remediated rapidly by visually-based movement-discrimination exercises, significantly improving cognitive abilities, so that a person’s quality of life improves rapidly, when other methods have been unsuccessful. We will show that a paradigm shift for treating visual timing deficits found in a wide range of different of cognitive disorders is crucial.

The movement-discrimination intervention used in this study is believed to improve the precision in timing of visual events, and thus accelerate reading progress by increasing processing speed, selective and sustained attention, and working memory span (16–20). It achieves this by improving the function of the dorsal stream, boosting magnocellular relative to parvocellular activity, thereby improving inhibitory and excitatory circuits, in feedforward and feedback pathways, taking advantage of the brain’s neural plasticity (21). Visually-based movement-discrimination exercises in both normal participants (16, 22–27), dyslexics (14–16, 18, 19, 25), and after a TBI (15) have demonstrated neuroplasticity in domain of processing speed by practicing these exercises over a short period of time. These studies found that the more movement-discrimination was practiced, the more motion sensitivity, attention, memory, and reading skills improved, indicating that timing deficits are a key factor preventing normal cognitive function in dyslexia, aging, and after a TBI. Movement-discrimination exercises were not only more effective, but also were completed 2–8 times faster than other reading and cognitive interventions (14). We provide additional evidence showing that reading, attention, processing speed, problem solving, and memory problems involve neural timing deficits in visual system’s dorsal stream. These deficits in the dorsal stream affect both feedforward and feedback pathways between visual, parietal, and frontal areas.

The visual system has been hypothesized to exploit the dichotomy of a fast magnocellular channel (dorsal visual stream) together with a slower parvocellular channel (ventral visual stream) for the purpose of selective attention (28–30). The major dorsal stream attentional pathway, receiving predominantly magnocellular input is specialized for processing the location and movement of objects in space, whereas the ventral stream receives both magnocellular and parvocellular inputs and is specialized for extracting the details related to an object’s color and shape (31–33).

The dorsal visual stream provides the input to the attention networks (28–30). The control of spatial attention in early visual cortex is likely to be directed by regions of the Posterior Parietal Cortex (PPC) and dorsal lateral Prefrontal Cortex (dlPFC) (28–30, 34–37). Top-down attentional feedback occurs in the PPC where increased gamma activity is shown to be linked to visual attention and planned saccadic eye movements (38). The parvocellular neurons in the ventral stream subsequently use the coupled alpha-gamma oscillations regulated by the pulvinar for sequential processing (39), as a starting point for deciphering the individual letters (18, 19, 28–30, 40, 41). Sequential processing also uses the functional anatomy of the claustral connections of items being processed serially, such that cross-frequency coupling between low frequency (theta) signals from the claustrum and higher frequency oscillations (gamma) in the cortical areas is an efficient means for the claustrum to modulate neural activity across multiple brain regions in synchrony (42). The timing, period, envelope, amplitude, and phase of the synchronized coupled theta-gamma oscillations are modulating the incoming signals to the striate cortex, and have a profound influence on the accuracy and the speed of reading (30). It is likely that the dyslexic reader’s deficit in attentional focus (43, 44) is a consequence of slow magnocells preventing the linked parvocellular neurons from being able to isolate and sequentially process the relevant information that is needed for reading (28, 29, 45). Cross-frequency theta-gamma coupling enables sensory areas of the brain which capture language stimuli to communicate rapidly with higher-order brain areas for real-time processing of language input (38, 46), playing a crucial role in mediating working memory and in enabling learning (47, 48). Both claustral connections (42) and the pulvinar complex (39) regulate synchronous information transmission between cortical areas based on attentional demands.

Contrast sensitivity-based movement-discrimination training employing figure/ground discrimination improved not only magnocellular function and attention, but also improved magno-parvo integration, figure/ground discrimination, and feedback measured by the strength of coupled theta/gamma activity for the test patterns moving at 6.7 and 8 Hz and coupled alpha/gamma activity for test patterns moving at 10 and 13.3 Hz (14, 15, 18, 19, 25, 27). Moreover, feedback in the dorsal stream from middle temporal cortex (MT), the specific cortical region vital for movement discrimination (31), to V1 improves figure/ground discrimination (49) a task used when reading by discriminating the letters in the word from the remaining text, or discriminating direction of movement relative to a background (50). Furthermore, feedback from MT has its strongest effects for low salience stimuli (49), such as low contrast patterns having less than 10% contrast, i.e., those patterns that maximally activate magnocellular neurons (51, 52). When movement-discrimination training was done using patterns optimal for activating the V1-MT network (49, 53, 54) visual timing deficits were remediated for those with a TBI, causing attention, reading fluency, processing speed, and working memory, all high-level cognitive functions, to improve significantly (15). These results were also found for those with dyslexia (14, 19, 27, 50) and older adults (55).

The scientific premise for using contrast sensitivity-based movement-discrimination training is that remediation of a fundamental visual timing deficit affecting motion discrimination at a low level of cognitive processing generalizes to high level cognitive skills (attention and working memory) reliant upon motion processing as a foundation. Sluggish motion cells make it difficult to locate the beginning and end or identify the order of letters in a word, causing confusion, mis-sequencing, and hence slow reading. Thus, slow neural pathways cause the brain to misdirect visual attention, confuse what the eye sees, and reduce the ability to remember the visual forms of words (14, 15, 19, 27). The movement-discrimination training enhances coupled theta/gamma and alpha/gamma oscillations, improving both the feedforward and feedback attention and executive control networks conveyed by the dlPFC and PPC to modulate attention in MT and striate cortex (V1), enabling a wide range of cognitive skills to improve. This theory of change is validated further by the results of this study: MEG imaging showing the improvements in these networks, and the improvements in cognitive skills found following short period of movement-discrimination training. Contrast sensitivity-based left–right movement-discrimination exercises is the first visually-based intervention that was found to improve both low-level movement-discrimination in the dorsal stream and high-level cognitive functioning. This has been demonstrated both behaviorally and using MEG brain imaging, improving the attention and executive control networks in dyslexics (14, 56) and after a TBI (15). Since contrast sensitivity-based movement-discrimination neurotraining is so rapid and effective, it offers a new approach that represents a paradigm shift in the treatment of dyslexia, one that is based on improving visual timing instead of targeting higher level phonological timing (14, 19).

MagnetoEncephaloGraphy (MEG) brain source imaging, providing a neural correlate, was conducted to determine the brain areas that increase in function for an adult dyslexic following these left–right movement-discrimination exercises. This neural correlate for dyslexia shows for the first time that these left–right movement-discrimination exercises improve the function of the motion area: MT in the first 300 ms, improving the sensitivity and neural timing of magnocells in the dorsal stream. Improvements in cognitive skills were also measured for an older adult with a battery of neuropsychological tests of cognitive skills, for the first time.

## 2. Methods

### 2.1. Participants

A 29 years-old dyslexic Caucasian man answered an ad to improve cognitive skills that was posted by UCSD. He had been finding his quality of life was limited by his dyslexia and was interested in any methods to improve his cognitive skills. A healthy 71 years-old Caucasion woman, having a PhD in physics, referred by a professor at UCSD, enrolled in this study, since she wanted to improve her ability to remember. Lately, she found her vision and memory were not as reliable, increasingly reducing her quality of life, especially when driving during dawn or dusk.

The subjects had tried many different interventions that were all unsuccessful. When asked, neither subject could recall the names of these interventions. These vision and cognitive deficits were not experienced by other family members, so not likely to be genetic in origin. Behavioral pre-tests, shown in Tables 1, 2, confirmed both subjects concerns about their cognitive abilities. The inclusion criteria consisted of: wanting to improve their visual and cognitive skills, agreeing to complete two sessions of PATH training twice a week for 8 weeks, at the same time of day, around 11 am, so they were not tired, and agreeing to follow PATH training with at least 30 min of cognitive exercises. Since there was no control condition, there was no blinding in this study.

**Table 1:** Dyslexic pre-post standardized percentiles and reading scores.

Standardized tests	Pre-test	Post-test
Reading speed	154 wpm	437 wpm
IVA+ focusing attention	1%	54%
IVA+ sustained attention	10%	82%
IVA+ impulsivity	18%	62%
WAIS processing speed	23%	50%
TIPS visual working memory	6%	99%
TIPS delayed recall	1%	25%

**Table 2:** Older adult pre-post standardized percentiles and reading scores.

Standardized tests	Pre-tests	Post-tests after 8 weeks	Post-tests 4 years later
ADT visual processing	Markedly Below Normal	Above Normal	Above Normal
ADT phonological processing	Mildly Below Normal	Above Normal	Above Normal
WRAT reading	75%	87%	87%
WRAT spelling	58%	61%	73%
WRAT math	73%	95%	92%
Reading speed	229 words/min	541 words/min	430 words/min
WAIS processing speed	42%	77%	87%
DKEFS attention	81%	87%	81%
DKEFS cognitive flexibility	81%	87%	81%
TIPS visual working memory	34%	86%	87%
WAIS auditory working memory	55%	97%	90%

Both participants, who lived in the San Diego area, signed informed consent forms, the dyslexic subject’s consent form approved by UCSD Institutional Review Board, and the older adult’s consent form approved by SolutionsIRB, a full service private Institutional Review Board registered with OHRP. This informed consent form assured the subject their data would be anonymous. This study was conducted in accordance with the Declaration of Helsinki, and the protocol was approved by each IRB. The intervention exercises were conducted in a room devoted to this task at either UCSD, Perception Dynamics Institute, or once learned at home.

### 2.2. Intervention: visual movement-discrimination task- PATH to Reading™ (PATH) neurotraining

**Figure 1:**
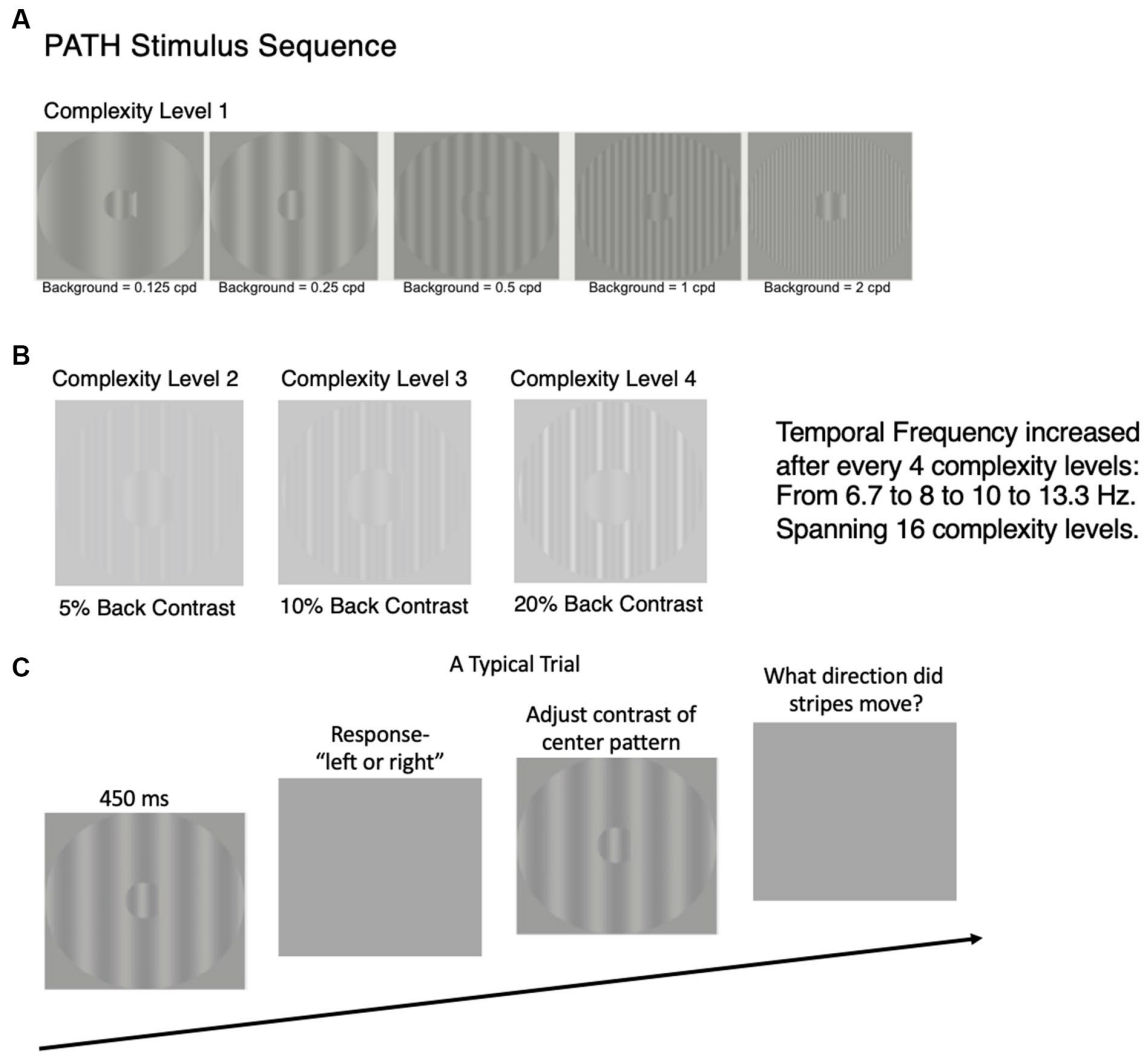
Time sequence for study design. After subjects were enrolled in study, a battery of visual and cognitive skills standardized tests were administered at the beginning and end of this study, results reported in Tables 1, 2. After standardized tests were administered, the PATH neurotraining program was administered for 8 weeks. The details of the PATH neurotraining program are presented in **(A–C)** and in the video (https://youtu.be/HgCZn9uVdS0). **(A)** Sample patterns for intervention at Complexity Level 1 for a background two octaves lower in spatial frequency than the test frequency, one octave lower in spatial frequency than the test frequency, equal in spatial frequency to the test frequency, one octave higher in spatial frequency than the test frequency, and two octaves higher in spatial frequency than the 0.5 cyc/deg. “fish shaped” test pattern. This same set of backgrounds was presented in this order for each of the 4 test spatial frequencies (0.25, 0.5, 1, and 2 cyc/deg). **(B)** Complexity levels 2, 3, and 4 display multifrequency backgrounds for center pattern in 1.A (0.5, 0.5 cyc/deg), having the same fundamental frequency as in complexity level 1, with a difference frequency equal to the test frequency, increasing the background contrast from 5 to 10 to 20% contrast. **(C)** A typical trial for *PATH to Reading* /Insight intervention. Pattern flashes on screen for <= 450 ms while center stripes move left or right. Screen goes blank, waits for left or right arrow key to be pushed. If incorrect, short tone sounds. As soon as left or right key pressed, next pattern with same or different contrast flashes on screen while center stripes move left or right. This sequence of patterns is presented continuously until the contrast threshold for this pattern is measured (20–40 trials). At the end of each contrast threshold measurement, a fishnet appears with a fish for each pattern having a contrast threshold <= 1% contrast, personal best score, current score, and number of patterns remaining. Then the next pattern combination is presented to measure next contrast threshold until all 20 *PATH neurotraining* patterns were presented, and the program says ‘Thank You’, presents a star for each level of complexity completed, shows a graph with the contrast threshold function (optimal, current, initial) for each test frequency with its 5 background patterns, and quits.

The patented (16, 17) visual timing intervention^1^ uses dim grayscale patterns optimal for activating magnocellular (magno) neurons to retrain the brain’s pathways (15–19), see Figure 1 and https://youtu.be/HgCZn9uVdS0. These patterns are designed to activate motion pathways (by using left–right movement) relative to the pattern pathways by using a stationary background that entrains motion discrimination (23, 24). Each pattern is presented for less than half a second, increasing from slow theta movement (6.7–8 Hz) to faster alpha movement (10–13.3 Hz) every 4 complexity levels. Only the contrast of the center stripes (the test spatial frequency) in the fish shaped pattern that moves left or right relative to a stationary striped background is dimmed until the direction can no longer be seen. The low contrast (0.1–5%) test frequency was set to either 0.25, 0.5, 1, or 2 cyc/deg., being an octave apart. The five stationary vertical background gratings for each test frequency bracket the test frequency, having a fundamental frequency equal to the test frequency or ±1 or 2 octaves from it. Only when movement-discrimination is done relative to a structured background, do all types of dyslexics exhibit motion discrimination deficits (18, 19, 25, 26).

The subject sat 57 cm in front of a 13-inch MacBook Pro computer monitor, with a display similar to the ones in Figure 1. During the presentation, the bars in the “fish-shaped” window in the center of the screen formed by a sinusoidal grating, moved left or right very briefly (<= 450 ms). The fish-shaped pattern subtended 4 deg. visual angle, and the structured background subtended a 16 deg. visual angle. When the screen went blank, the subject reported which way the center pattern moved by pushing the left or right arrow key (Figure 1C). A brief tone was presented after incorrect responses. The program adaptively changed the contrast of the test pattern in order to keep the subject at 79% correct. There are also levels of difficulty introduced by making the background pattern more similar to that in the fish, see center pattern in Figure 1A, and by increasing the pattern’s complexity level (Figure 1B). The complexity level increases: (1) the number of sinewave components in the background from one (Figure 1A) to three harmonically related frequencies having a difference frequency equal to the test frequency (Figure 1B), shown previously to facilitate movement discrimination (23, 24) by providing a wider background frame of reference, (2) the background contrast from 5 to 10 to 20%, to increase the amount of parvocellular activity, since magno-cells saturate at 10% contrast (51), and (3) the pattern’s speed of movement after every 4 complexity levels, increasing from 6.7 to 8 Hz (in theta range) to 10 to 13.3 Hz (in alpha range), so that the subject was challenged as the training progressed. Faster speeds of movement, 10 Hz to 13.3 Hz, were too fast to be trained on until slower speeds of movement had been trained.

At the start of a session, both the test and background gratings were set to 5% contrast to ensure that the contrast of the test pattern is in the middle of the magnocellular contrast range (51). The mean luminance was approximately 120 cd/m^2^, measured using a Pritchard 1980A Spectra photometer. Each time the subject correctly identified the direction the fish stripes moved, the contrast of the test grating was lowered one step until the subject answered incorrectly. Following the first incorrect response, a double-staircase procedure (22) was used to measure the movement-discrimination contrast threshold. Lowering a subject’s contrast threshold is what increased a subject’s sensitivity to motion discrimination. This staircase procedure estimates the contrast threshold by using the most sensitive, repeatable measurements of contrast sensitivity possible (57). Each contrast threshold required 20-40 trials. A full training cycle of the movement-discrimination task required 20 contrast threshold determinations: for each of the four test spatial frequencies (0.25, 0.5, 1, and 2 cyc/deg) paired with each of the five background spatial frequencies (equal to test frequency or ±1 or ±2 octaves from the test frequency), patterns chosen to optimally active magno-cells, see Figure 1A. After each contrast threshold measurement, a score was given to make the training more game-like. The lower the contrast threshold, the higher was the score. Other motivational strategies in PATH training included earning a fish for each low contrast threshold (<= 1% contrast), showing a graph at end of each training cycle displaying original, current, and optimal contrast sensitivity function for each test frequency, and a star for each complexity level completed. This training was adaptive in response to the subject’s performance, and incorporates cycles of feedback and reward at multiple levels, ranging from positive and negative feedback on a trial-by-trial basis, as well as cumulative block and session feedback. Such feedback greatly accelerates learning (58, 59). This interactive training procedure (15–19, 25, 26) and feedback motivated the user to continue to improve. Motion direction-discrimination was trained for between 15 and 20 min to complete one training cycle, twice a week for 8 weeks. Initially this intervention was administered one-on-one by staff. After the intervention was learned, it was completed by each subject at their home unsupervised. This training was followed by at least 30 min of reading an interesting story (dyslexic) or cognitive exercises (older adult), helping this training to generalize to high-level cognitive skills.

#### 2.2.1. Fidelity of implementation

Contrast threshold data was collected using the most sensitive, repeatable measurements of contrast sensitivity (57). All contrast threshold data with date and time stamps was stored in individual and summary files, and collected automatically by the computer. Therefore, there was no means for tampering with the data collection. Data in summary files showed each subject’s contrast thresholds, and how long it took to complete each threshold. This summary data was examined weekly to ensure the subject was completing one training cycle twice a week and seeing left–right movement dimly. Compliance was never a problem.

### 2.3. Neural correlate: MEG source imaging

Two MEG exams were performed for the dyslexic subject: one before and another after 8-weeks of the movement-discrimination intervention to evaluate whether he had significant improvements in brain functioning after intervention training. A structural MRI used for superimposing the functional activity on top of the brain anatomy was done before initial MEG recording. MEG responses evoked by a 2.5% contrast 1 cyc/deg. sinewave grating moving left or right at 10 Hz relative to a 5% contrast 1 cyc/deg. background, using the same time sequence, as described above and in Figure 1C, was collected using the VectorViewä whole-head MEG system (Elekta-Neuromag, Helsinki, Finland) with 306 MEG channels. The movement-discrimination task entails on-line monitoring, updating, and manipulation of remembered information. During this task, the subject was required to monitor the direction of movement. A fixation cross was presented during the 3,000 ms interstimulus interval. The subject was instructed to push a right button if the test pattern moved right relative to the background and push a left button if the pattern moved left relative to the background. About 50 trials per load condition were collected for this subject. Performance was recorded using an MEG-compatible response pad, in which index finger blocks-and-unblocks a laser-beam.

The dyslexic subject, who did not have metals objects in his brain, was seated in an upright position inside a multi-layer magnetically-shielded room at the UCSD MEG Center. MEG data were sampled at 1000 Hz and were run through a high-pass filter with a 0.1 Hz cut-off, and a low-pass filter with a 330 Hz cut-off. Eye blinks and eye movements were monitored using two pairs of bipolar electrodes with one pair placed above and below the left eye, and the other pair placed on the two temples. Heart signals were monitored with another pair of bipolar electrodes. Precautions were taken to ensure head stability; foam wedges were inserted between the subject’s head and the inside of the unit, and a Velcro strap was placed under the subject’s chin and anchored in superior and posterior axes. Head movement across different sessions was about 2–3 mm on average. Analysis of MEG sensor waveforms were described previously (15, 60–62).

### 2.4. Behavioral measures used before and after intervention (pre-post tests)

Improvements in cognitive skills were measured using a battery of neuropsychological tests, described below, administered by trained staff before and after intervention training in the middle of the day around noon so subject was not tired or hungry. These tests were chosen since they are considered the gold standard for assessing impairments in cognitive function (15). Based on subject’s raw score and age, a standard score that was converted into a standardized percentile score was assigned. Visual skills were measured using tests of near visual acuity (Good-Lite acuity card held 16 inches away), and measuring the eyes’ convergence near-point (distance where 1 cm letter ‘A’ blurs or becomes two).

The tests of cognitive skills that were completed are:

1. Adult Dyslexia Test (ADT), https://www.good-lite.com/products/482700, to measure reading proficiency evaluated whether a subject’s visual processing and phonological processing was above normal, normal, borderline, mildly below normal, moderately below normal, or markedly below normal.
2. Wide Range Achievement Test (WRAT) measures Reading: number of words read correctly, Spelling: number spelled correctly, and Math: number math problems solved correctly in 15 min.
3. Computer-Based Reading Speed test determined speed needed to read six consecutive words in story, *Wrinkle in Time* by Madeleine L’Engle, on computer screen, at increasing speeds to measure two reading rate thresholds (16–19, 25–27, 63).
4. Attention tests measured using either: Integrated Visual and Auditory Continuous Performance Plus (IVA + Plus) Tests from *BrainTrain* to measure attentional focus, sustained attention, or inattention, hyperactivity, and impulsivity by measuring the accuracy and reaction time of different responses to different tasks that are scored and converted to standardized percentile scores by the IVA+ computer program, Or Delis-Kaplan Executive Function System (DKEFS) Color-Word Interference test, where subject says printed color of words that denoted a different color (Stroop Attention test), and Attention Switching (Cognitive Flexibility), switching between color of word and what word says when surrounded by a rectangular box. The standardized percentile is reported in Tables 1, 2 (Attention and Cognitive Flexibility) since these are the most meaningful scores to understand attention levels pre and post intervention training.
5. Wechsler Adult Intelligence Scale (WAIS)-4 Processing Speed required two subtests: (1) the WAIS Symbol Search subtest which required subjects to scan a target group (two symbols) and search a group of 5 symbols, indicating whether one of the target symbols appeared in the search group, and (2) WAIS Digit Symbol Coding subtest, where the subject filled in boxes below digits with symbols that were paired with them in a key at the top of the page. Both of these subtests were timed for 2 min each. The scaled scores from each subtest were combined to create an overall Processing Speed Index score, that was converted to a standardized percentile score.
6. WAIS-4 Working Memory Index to measure Auditory Working Memory (AWM) required two subtests: (1) the Digit Span subtest, where the subject had to repeat a list of spoken numbers, requiring the subject to remember subsequently more numbers: in the correct order, backwards, and in numerical sequence on three different subtests, and (2) the Letter-Number Sequencing subtest which required sequencing subsequently more numbers and letters in the correct numerical and alphabetic sequence. Presentation of the numbers and letters were timed for one second each for these working memory tests.
7. Visual Working Memory (VWM) using the Test of Information Processing Skills (TIPS), provided by WPS: *WesternPsychologicalServices*. The subject recalled a sequence of letters presented visually one at a time for 2 s each, for sequences of from 2 up to 9 letters right after seeing the entire sequence of letters. Short Term VWM was assessed by recalling the correct sequence of letters after counting from 1 to 10 numbers in sequence, starting at different initial numbers, slowly, and after repeating a short sentence with an animal subject for VWM. Delayed Recall was assessed by remembering all animal names in repeated sentences 3 min after finish the VWM test.

All of these cognitive assessments, which were age-appropriate, took 1.5 h to complete.

## 3. Results

**Figure 2:**
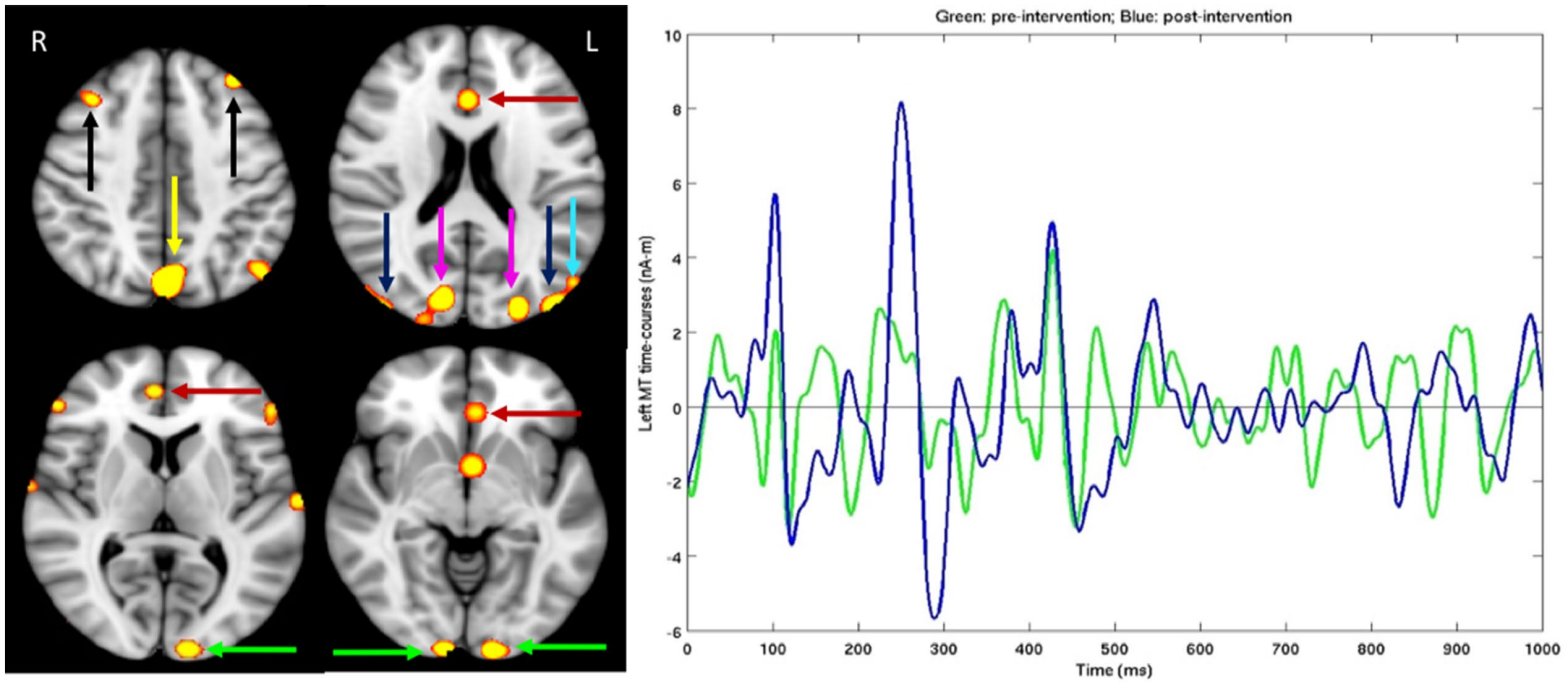
For dyslexic subject, after standardized tests were administered MEG brain imaging before and after the PATH neurotraining intervention was completed. Left Panel: Different slices from the left (L) and right (R) sides of the brain. Hot spots showing significant MEG source magnitude (i.e., root-mean-square measure) signal increases were computed from mean square measure of MEG signal increases for the 0–1,000 ms time interval following the stimulus onset in post- versus pre-intervention exams, for an adult male dyslexic aged 29 years. Familywise error was corrected for multiple comparison across spatial voxels using standard cluster analysis. For those hot spots, the corrected value of *p* thresholds were at *p* = 0.01 for red, and *p* = 0.001 for bright yellow color. Green arrows: Visual Area 1 (V1); Blue arrows: Middle Temporal (MT) cortex; Magenta arrows: visual Area 3 (V3); Cyan arrow: Medial Superior Temporal (MST) cortex; Red arrows: Anterior Cingulate Cortex (ACC); Yellow arrow: precuneus/Posterior Cingulate Cortex (PCC); Black arrows: dorsal lateral Prefrontal Cortex (dlPFC); Right Panel: MEG source time-courses from left MT area during post-intervention (Blue line) and pre-intervention (Green line) exams in the figure above.

Both MEG brain imaging, see Figure 2, and neuropsychological behavioral tests, see Tables 1, 2, found substantial improvements in the visual, attention, and executive control networks after PATH to Reading™ training. The dyslexic subject read *A Wrinkle in Time* for 30 min after each training cycle (2 sessions), whereas the older adult practiced cognitive exercises, either math problems or playing chess, for 30 min following each training cycle, since previous research (14) found that practicing on what one needs to improve is essential for PATH neurotraining to be effective. Both subjects found the intervention to be easy to complete, noticing the improvements right away, and had no trouble completing the intervention on their own, after initial training at UCSD or Perception Dynamics Institute.

### 3.1. Dyslexic adult improved in reading, attention, processing speed, and memory

MEG brain imaging, Figure 2, right panel, shows that improvements in MT (difference between pre-and post- MEG exams) happen in the first 300 ms. Showing that this training sped up magnocells in visual dorsal pathways. The details of the evoked potentials in MT are shown (right panel), whereas the data from other regions were not chosen, because MT is the main region of interest for improving the function of the dorsal stream that has been shown previously not to be responsive in dyslexics (64, 65). The improvements in MT reveal improvements in P1 and N1, showing that the post-intervention signal is increased in sensitivity for both excitatory and inhibitory signals, at durations between 80 and 150 ms that result from *alpha activations* (10–13.3 Hz), and in P2 and N2 at durations between 240 and 300 ms from *theta activations* (6.7–8 Hz), improvements in N1 and N2 known to be enhanced by selective attention (66) were found for older adults following twice as much perceptual training (67). Different slices from the left (L) and right (R) sides of the brain are shown in the left panel since the brain exhibits asymmetrical functioning in the two sides of the brain, with dyslexics showing more cognitive deficits in the left side of the brain (68). Hot spots showing significant MEG source magnitude (i.e., root-mean-square measure) signal increases were computed from mean square measure of MEG signal increases for the 0–1,000 ms time interval following the stimulus onset in post- versus pre-intervention exams, for an adult male dyslexic aged 29 years. Familywise error was corrected for multiple comparison across spatial voxels using standard cluster analysis. For those hot spots, the corrected value of p thresholds were at *p* = 0.01 for red, and *p* = 0.001 for bright yellow color. Substantial MEG signal increases (left panel) in visual Motion Networks [V1, V3, MT, Medial Superior Temporal cortex (MST)], Attention Networks [Anterior Cingulate Cortex (ACC) and precuneous/Posterior Cingulate Cortex (PCC) areas] and Memory Networks (dlPFC) were observed following a short period of training on contrast sensitivity-based movement-discrimination, the same improvements found after a TBI (15).

Behavioral tests, shown in Table 1, found that his reading speed improved almost 3-fold, and his processing speed improved from much lower than average (23%) to average (50%). Moreover, his attention skills improved markedly. His performance in IVA+ Focusing Attention improved from the lowest 1% to just above average (54%), his IVA+ Sustained Attention improved from the lowest 10% to 82% (i.e., better than 82% of his peers), and his IVA+ Impulsivity improved from much lower than average (18%) to above average (62%). His working memory skills improved markedly as well. His visual working memory improved from the lowest 6% to 99% (better than 99% of his peers), and his delayed recall improved from the lowest 1% to 25%. He also improved in visual skills, markedly reducing his convergence insufficiency: his near point of convergence was reduced from 9 cm down to 3.5 cm and his visual acuity improved from 20/20 to 20/16. In addition to these improvements, his quality of life also improved remarkably, doing activities that previously were too overwhelming to consider: getting married, starting a business helping dyslexics, finishing college, and becoming an electrician, being very grateful. These improvements were retained years later, this subject reported.

These improvements in cognitive skills found for dyslexics following contrast sensitivity-based movement-discrimination training were not found by targeting higher level phonological skills like FastForWord, nor linguistic-based training like Learning-Upgrade (19), nor computer-based repeated reading (14, 27, 69) which was 4-fold less effective. These improvements were also found in 6–8 year-old typically-developing children (25–27, 50), which is the age when the temporal lobe shows peak synaptogenesis (70). These findings support the hypothesis that visual magnocellular pathways provide the gateway for attentive processing (28–30) and reading (71–73), since timing impairments can be reduced following training using contrast sensitivity-based left–right movement-discrimination exercises (19, 27). Research finds that there is an imbalance between magno- and parvo-cellular systems in dyslexics (74). These results confirm the causal role of visual motion sensitivity and faulty synchronization of parvocellular with magnocellular visual pathways in the dorsal stream as a fundamental cause of dyslexic reading problems.

### 3.2. Older adult improved in reading, attention, processing speed, and working memory

**Figure 3:**
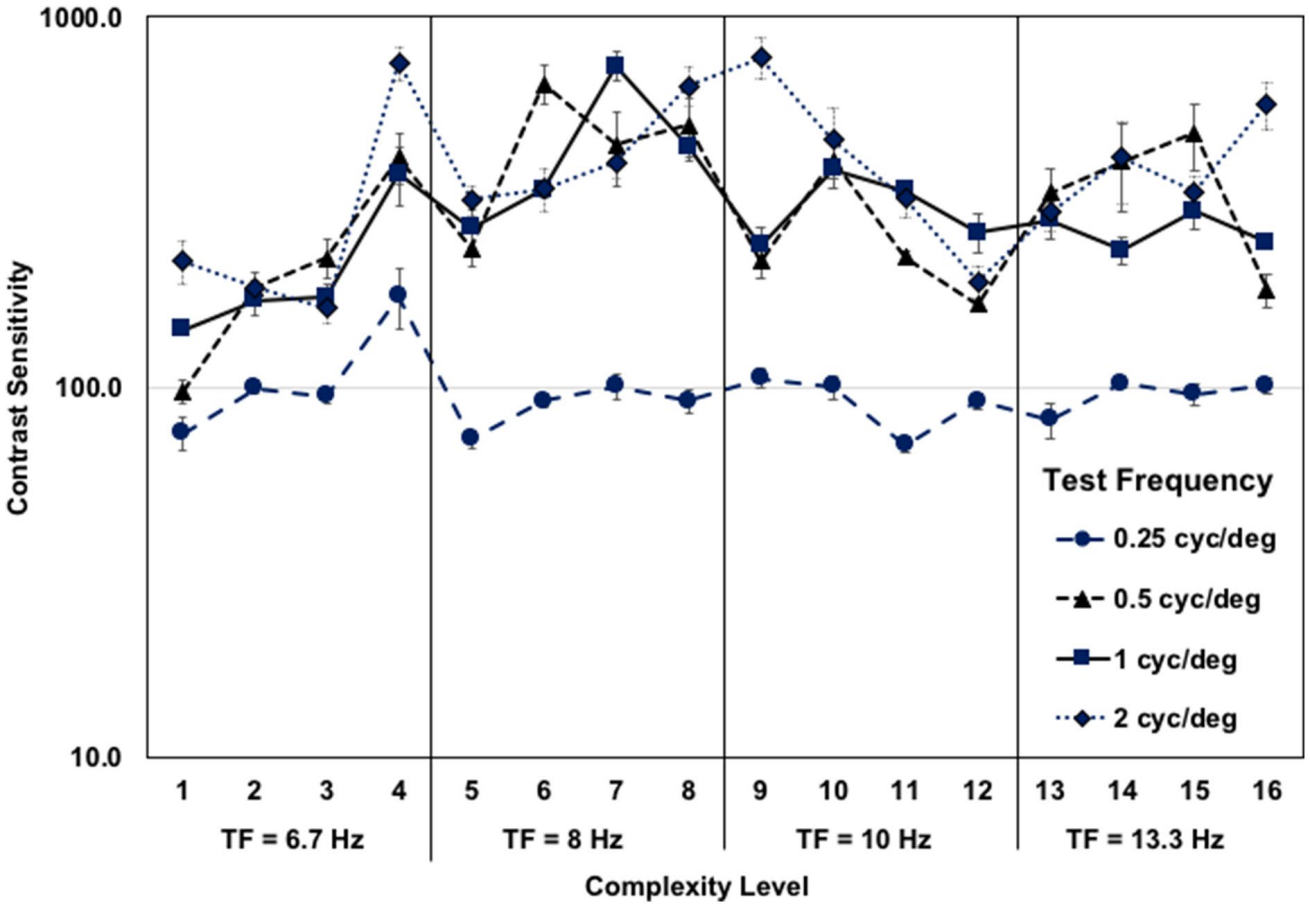
Older adult mean contrast sensitivity function for left–right movement discrimination at each complexity level, for each test frequency, when averaged across the five background patterns, each 4 complexity levels having a faster test frequency temporal frequency (TF). The first eight complexity levels trained coupled gamma/theta oscillations (6.7–8 Hz) and the second eight complexity levels trained coupled alpha/gamma oscillations (10–13.3 Hz), showing similar contrast sensitivities for both.

The older adult’s contrast sensitivity function for movement-discrimination for each test frequency, when averaged across the five background patterns at each level of complexity, is shown in Figure 3. These contrast sensitivities are amongst the highest yet recorded, showing age was not a limiting factor. The contrast sensitivities for the widest bars, 0.25 cyc/deg., were much lower than for other test frequencies, supporting the hypothesis that information from several spatial-frequency neural channels must be combined for movement-discrimination, making this task so difficult. The highest contrast sensitivities were found for 2 cyc/deg. test frequency, detected using a single spatial-frequency channel (75). A similar pattern of contrast sensitivities for movement-discrimination in older adults was found previously (55).

After 8 weeks of movement-discrimination training, and practicing cognitive exercises, either math problems or playing chess, for 30 min following each training cycle, large improvements in working memory (VWM: from 34 to 86%, AWM: from 55 to 97%) were found for the older adult who was already adept at paying attention, yet still improved after training, see Table 2. Moreover, her processing speed improved from 42 to 77%. Her reading skills improved markedly, more than doubling in reading speed, dyslexia improving to above normal, and WRAT Reading, Spelling and Math improving substantially, as shown in Table 2. These results show that not only reading, attention, processing speed and working memory skills, but also problem-solving skills improved markedly, showing cognitive transfer to untrained tasks. When this older adult was tested on these behavioral tests 4 years and 2 months later, when she was 76 years old, having had no additional cognitive training, the remarkable cognitive improvements were still evident, showing that the improvements in cognitive skills after PATH training are sustained over time. Remarkably, some cognitive skills were even higher 4 years later, see Table 2, including processing speed, now 87% (improving 10% more), VWM, now 87%, and Spelling now 73%. AWM was still high at 90%, as was problem solving at 92%. Not only cognitive skills improved, but her visual skills also improved, improving from a near point of convergence of 25 cm down to 15 cm. Her visual acuity of 20/32 did not change. These results validate the reported improvements in cognitive skills found previously (55) using robust neuropsychological tests. Since coupled alpha-gamma activity is reduced in older adults with mild cognitive impairments (76), these improvements in processing speed and working memory provide more evidence movement-discrimination training improves coupled alpha-gamma activity.

Following contrast sensitivity-based left–right movement-discrimination training, this older adult’s quality of life improved remarkably. She reported “My memory, reading speed, ease of understanding, processing speed, ability to multitask, concentrate, and pay attention have improved remarkably in just a few months. Since doing PATH training, I find that driving is much easier, and I am able to attend to a much wider region, allowing me to see street signs more easily. I am now able to distinguish the other car movements at dawn and dusk much better improving my driving skills. These improvements in remembering, concentrating, and reading have made life much easier and more enjoyable. I have noticed that everyday activities are so much easier to complete and are more enjoyable. I hope that you are able to help other older adults so that forgetting and everyday activities are no longer difficult.” This subject reported that these improvements have not degraded over time, and are really appreciated.

These results demonstrate neuroplasticity in the domain of visual motion sensitivity and timing, processing speed, reading speed, attention, working memory, and problem solving using a short period of practice on discriminating moving patterns that optimally activate magnocells, relative to a stationary background that optimally activates parvocells. We found, using MEG source imaging and behavioral neuropsychological tests, that speeding up these motion cells improves not only the visual pathways, but also the attention and executive control networks of both young adults who are dyslexic, and older adults with mild cognitive impairments. The dramatic improvements are reliable and cannot be due to practice effects, since none of the test items nor their order of presentation could be memorized. These improvements are also verified by patient reports at the end of the study and years later. Subjects experienced no adverse effects, only benefits.

## 4. Discussion

This study supports our working hypothesis (14, 15, 19, 27) that magnocellular neurons in dorsal visual pathway (V1-MT) of dyslexics are sluggish, causing visual timing deficits at lower levels of visual processing that disrupt processing at higher levels of dorsal stream processing, including the development of these visual, attention, and executive control pathways. Considerable evidence confirms that many dyslexic readers demonstrate impairments in motion perception that rely upon magnocellular functioning. People with dyslexia have been found to have motion perception deficits at each of the processing levels in the magnocellular stream (14). These visual timing deficits limit reading acquisition in dyslexics. These results suggest a strong relationship between dorsal stream processing and reading ability, such that poor dorsal stream processing caused by sluggish magnocells is associated with slower timing and poorer reading skills (14, 16–20, 25–29, 63, 71–73, 77–80). Dyslexics lack the ability to process sequential information quickly and accurately, causing deficits in both reading speed and comprehension. These findings show that just by doing rapid brain exercises that improve a person’s ability to discriminate left–right movement relative to a stationary background pattern, improving the brain’s timing, one’s ability to read rapidly and accurately can be improved significantly.

These improvements indicate that remediation of visual timing deficits (visual motion networks), via PATH training, generalizes to high level cognitive abilities, improving the function of not only the hubs of the attention networks (ACC, precuneus/PCC) but also the hub of the executive control network, dlPFC, where working memory is analyzed (81). Notice that the left cortical areas V1 and MT showed more improvements than the right V1 and MT, which is consistent with previous imaging studies showing that dyslexics have reduced activations in the left temporal, parietal, and fusiform regions (68). These improvements in visual, attention, and memory networks are also validated behaviorally. Only by improving low-level skills (movement-discrimination) do high-level cognitive skills such as attention, processing speed, working memory and reading improve.

The MEG results from both an adult dyslexic and after a TBI (15) corroborate these findings, showing that the timing and sensitivity of magnocells in MT improve significantly after a short period of contrast sensitivity-based movement-discrimination training. Moreover, finding P1, N1, and P2, N2 MT signals improved markedly after only 5 h of training on movement-discrimination indicates that attentional signals driven by coupled alpha/gamma and theta/gamma oscillations, respectively, are enhanced (67). Furthermore, there is evidence that improvements in the cognitive skills of dyslexics after this movement-discrimination training that is more rapid and effective than the competition are sustained over time (14, 18, 19, 27), as also shown for the older adult in this study.

The data from this study provides new evidence that deficits in attentional focus, working memory, and navigation experienced by older adults result from timing deficits in the dorsal stream that are abated rapidly following training on contrast sensitivity-based movement-discrimination. Slower processing speeds and more effortful attention were found to explain a large part of age-related memory loss (82–84). This mental slowing can lead to inefficient processing based on strategies where further elaboration is required (85). Since this study found that improving bottom-up timing improved high-level cognitive skills, requiring coupled theta/gamma and alpha/gamma oscillations, this indicates bottom-up processing is the limiting factor in cognitive skills declining as we age. This conclusion is supported by the neural plasticity underlying visual perceptual learning in aging following training on a movement-discrimination task designed to activate MST (67). This perceptual training of older adults (67) produced large improvements in speed and accuracy, but no improvements in cognitive skills validated by neuropsychological tests, like those shown in Table 2, were reported. It is likely that contrast sensitivity-based movement-discrimination training, activating magnocells at both early and late levels of dorsal stream processing is more effective in improving cognitive skills in older adults.

### 4.1. Study limitations

This study has limitations since only two subjects were studied. The purpose of a case report is to demonstrate phenomena worthy of future investigation. Since those who completed PATH training, including dyslexics, older adults, and following a concussion experienced improvements in their visual and cognitive skills (14, 15, 17–20, 27, 50, 55, 56), we expect the improvements in visual and cognitive skills reported in this case report to be found when a larger group of subjects is studied. Additionally, due to sample size standard statistics showing whether these improvements are statistically significant cannot be assessed. Further limitations come from the relatively small number of trials in the imaging paradigm (approximately 200 at pre- and post- test, each). Future studies could improve the strength of the MEG signal with a larger number of experimental trials. However, the magnitude of improvements seen in the two cases presented are certainly promising. Currently, PATH training is used by some therapy centers, since some, like Stowell Learning Center, have found it rapidly remediates attention deficits that are not addressed by any other intervention. Future studies are planned to provide MEG neural correlates showing that coupled theta/gamma and alpha/gamma oscillations increase following PATH neurotraining.

## 5. Conclusion

Since this study found that a short period of movement-discrimination training improved dyslexic and older adult’s cognitive skills, both behaviorally and using a neural correlate, MEG physiological brain recordings, this data provides irrefutable evidence that improving low-level dorsal stream activity by increasing the timing and sensitivity of magnocells enhances coupled theta/gamma and alpha/gamma oscillations that enable improving both low- and high- level cognitive functions. Other cognitive training programs: (1) had little effect on improving the executive functions and attention in TBI (86, 87), (2) had results from brain training that were neither robust nor consistent, with transfer and sustained effects which were limited (88), and (3) improved only the task being trained on, and do not generalize to tasks not trained on or everyday cognitive performance (59). Currently, there are no proven solutions to improve attention and working memory in TBI patients (86, 89–91). We propose that rehabilitative treatments fall short because visual timing issues, persistent in individuals with a TBI (15, 92), with dyslexia (14, 17–20, 27–30, 50, 71–73), and older adults (55, 67, 82), are not being addressed. This study found that remediating visual timing deficits, via PATH training, generalizes to high level cognitive abilities not trained on, improving not only attention and memory, improving the functioning of the executive control network (81), but also processing speed and reading speed, behavioral measures of timing, after a short period of movement-discrimination exercises. By using a broad battery of pre-post neuropsychological tests, as well as pre-post MEG recordings, in this study and previously (15), the data indicate that PATH training improvements transfer to a broad range of cognitive abilities. These improvements were found for all users of PATH neurotraining (14, 17–20, 27, 50, 55, 56), also verified by testimonials on https://pathtoreading.com/testimonials. Recovering these cognitive skills will substantially improve a person’s quality of life. These studies indicate that a paradigm shift for remediating dyslexia and in treating attention, processing speed, memory, problem solving, and reading impairments in older adults, and after a concussion is needed.

## Data availability statement

The raw data supporting the conclusions of this article will be made available by the authors, without undue reservation.

## Ethics statement

The studies involving human participants were reviewed and approved by UCSD Institutional Review Board and SolutionsIRB. The patients/participants provided their written informed consent to participate in this study. Written informed consent was obtained from the individual(s) for the publication of any potentially identifiable images or data included in this article.

## Author contributions

TL and M-XH contributed to conception and design of the study. JS-T and M-XH performed the statistical analysis. TL wrote the first draft of the manuscript. TL, JS-T, and M-XH wrote sections of the manuscript. All authors contributed to manuscript revision, read, and approved the submitted version.

## Funding

This work was supported in part by Merit Review Grants from the U.S. Department of Veterans Affairs (P.I.: M.X.H., I01-CX002035-01, NURC-007-19S, I01-CX000499, MHBA-010-14F).

## Footnotes

^1^https://pathtoreading.com
